# Conceptual Framework for the Mapping of Management Process with Information Technology in a Business Process

**DOI:** 10.1155/2015/983832

**Published:** 2015-03-12

**Authors:** Vetrickarthick Rajarathinam, Swarnalatha Chellappa, Asha Nagarajan

**Affiliations:** ^1^Department of Management Studies, Anna University, Regional Office, Madurai 625007, India; ^2^Department of Management Studies, Fatima College (Autonomous), Madurai 625018, India

## Abstract

This study on component framework reveals the importance of management process and technology mapping in a business environment. We defined ERP as a software tool, which has to provide business solution but not necessarily an integration of all the departments. Any business process can be classified as management process, operational process and the supportive process. We have gone through entire management process and were enable to bring influencing components to be mapped with a technology for a business solution. *Governance, strategic management,* and *decision making* are thoroughly discussed and the need of mapping these components with the ERP is clearly explained. Also we suggest that implementation of this framework might reduce the ERP failures and especially the ERP misfit was completely rectified.

## 1. Introduction

The need of ERP is growing in a rapid phase one side but more than half of the implementations result in failure in other side. Many companies passionately go for the ERP with huge expectations, facing a lot of real-time issues to make it a grand success. Very recently, a term called misfit is often used by the ERP analysts and organizations. Misfit is the terminology representing the improper mapping of information technology with respect to business requirements. Misfit happens as ERP is the most complicated system to handle. Hence the solution for this concern emerged over the years. Scholars from different parts of the word presented articles on the reasons for misfit and tried to sort out the issue. The management process is one of the most important processes in the technology mapping and microanalysis of important elements is required. We discuss all the components of a management process which has to be mapped with technology to sort out misfit issues. This paper synergizes the various components, namely, management process, operational process, and supportive process of business by mapping with the technology paths via transactional database, business intelligence, web services, documentation, and workflow management. The corporate governance and strategic management are considered in this paper which are very critical implicational areas of ERP where the possibilities of misfit can occur. The SWOT matrix exhibited in this paper elucidates the compatibility and competency of the organizations to make the business solution a proper fit. The strategy formulated should be split up as corporate level, business level, and functional level; then only the uniformity will occur by applying the technology. This paper sorts out the strategy communication and its methods. To have a successful implementation of the formulated strategy, this paper emphasizes on [1] Scott Edinger's three Cs. To evaluate the strategy, the six-step decision making process SISCIC has been discussed in this paper. This paper describes the user group approach of ERP and its misfit issues.

## 2. Business

Business defined as an organization or economic system where goods and services are exchanged for one another or for money. Every business requires some form of investment and enough customers to whom its output can be sold on a consistent basis in order to make a profit. Businesses can be privately owned, not-for-profit, or state-owned.

## 3. Business Solution

Development of an end-to-end business solution mandates utilization of methodology that does not focus on departmental solutions or system (product) implementation. It needs to have a user-centric focus on implementing a manageable number of complementary technologies that support appropriate segments of the various key end-to-end business processes. These key business processes need to focus on adding value to the customers and incorporate a business strategy to expose required processes and information to customers and business partners. This information needs to come from the effective planned operational systems.

## 4. ERP System

**Figure 1:**
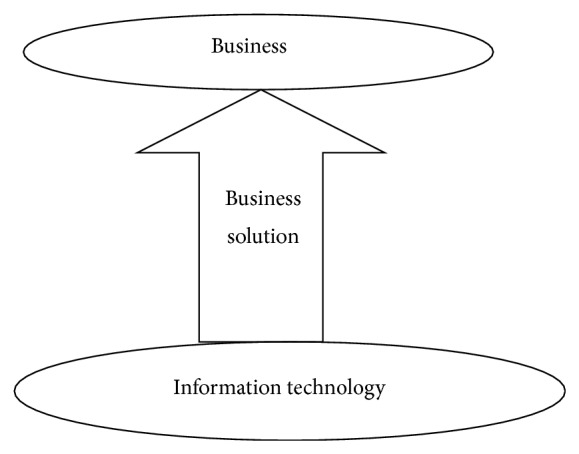
ERP system.

Actually the success percentage of an ERP system is the percentage of mapping between business and business solution. All the business solutions may not require integration of all the departments but integration can be a part of business solution (Figure 1).

Our definition for an ERP system is a software system which has to provide complete business solution to an organization.

## 5. Components of Business Solution

**Figure 2:**
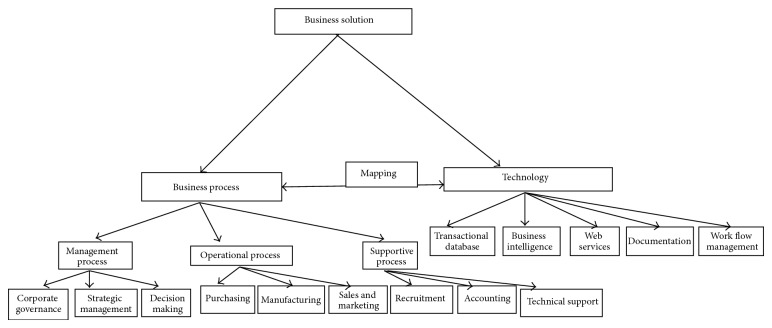
Business solution.

A good business solution is the mapping between business process and technology (Figure 2). Any business process can be divided in to three categories:

1. management process,
2. operational process,
3. supportive process.

### 5.1. Management Process

Management process is a process of planning and controlling the performance or execution of any type of activity. Corporate governance, strategic management, and the decision making are the key three components of any management process. The purpose of a management process is to ensure a disciplined and consistent approach to analysis and decision making. They facilitate the use of a logical thought process that is consistent with the objectives of the firm.

#### 5.1.1. Corporate Governance

Goal of an ERP business solution has to provide complete, accurate, and timely information flow system and produce much standard information for inside and outside stakeholders, making the management and stakeholder levels more transparent. At the same time, ERP implementation provides accurate and timely guarantee of information that enterprises disclose [2].

**Figure 3:**
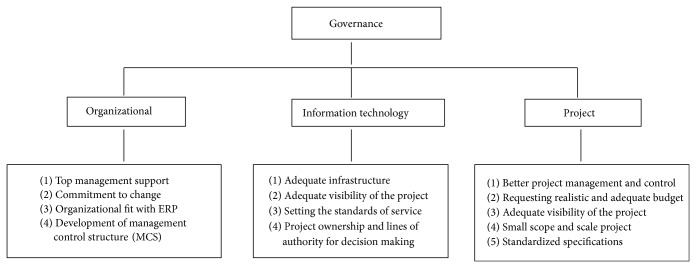
Governance.

*The Role of Corporate Governance in a Business Solution.* ERP solutions are actually business transformation projects, rather than straightforward large software development projects [3, 4] and their implementation has to change work processes and organizational structures, together with the daily activities of the majority of staff. Because of the business transformational nature, their failure is more likely to be due to organizational, social, or even political reasons than technical or software based causes [5]. Top management support is also important in each phase of development, from planning through project implementation and enhancement [6]. Governance is about providing strategic direction and planning and controlling projects and people and is delegated to project leaders (project governance), those responsible for IT (IT governance) and senior executives (organizational governance) by the Board of Directors (Figure 3).

#### 5.1.2. Strategic Management

A strategy is integrating organizational activities and utilizing and allocating the resources within the organizational environment so as to meet the present objectives. While planning a strategy, it is essential to consider that decisions are not taken in a vacuum and that any act taken by a firm is likely to be met by a reaction from those affected, competitors, customers, employees, or suppliers.

Ben Tregoe and John Zimmerman define strategy as “the framework which guides those choices that determine the nature and direction of an organization.”

**Figure 4:**
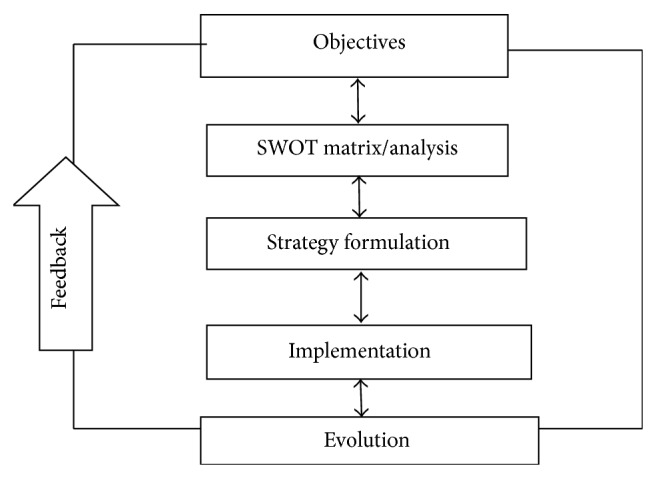
Strategic management process.

Strategic management is an ongoing process to develop and revise future-oriented strategies that allow an organization to achieve its objectives, considering its capabilities, constraints, and the environment in which it operates [7]. A key function of strategy is to provide coherence to the organizational action (Figure 4).

**Figure 5:**
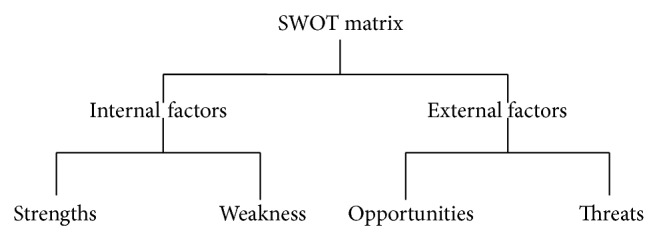
SWOT matrix.

*SWOT Analysis.* SWOT matrix is an analysis which is used to evaluate the strengths, weakness, opportunities, and threats of an organization's strategy. It is a structured planning method. Strength and weakness are the internal factors and opportunities and threats are the external factors. This is a tool for audit and analysis of the overall strategic position of a business environment (Figure 5). 

*Strengths.* This internal factor normally represents the organization's capabilities. Strengths can be tangible or intangible such as product, service, man power, and financial power.

*Weaknesses.* This internal factor normally represents the organization's obstacles towards achieving its objective. Example for weakness of an organization can be its quality, huge debts, insufficient man power, location, and so forth, but weakness of an organization can be controllable.

*Opportunities.* This external factor normally represents the planning and execution of new strategies that can be more profitable to the organization presented by the environment. Opportunities may be from market, competition, demand, government, and technology.

*Threats.* This is also an external factor that represents the condition which is hazardous to the organization's reliability and profitability. These threats are uncontrollable. Example for threats are increasing competition, market, unannounced power cuts, wars, and so forth.

**Figure 6:**
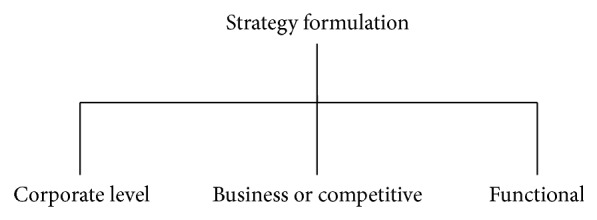
Strategy formulation.

*Strategy Formulation.* It is the appropriate steps needed to realize organization's mission and thereby achieving the vision of the organization. There are three aspects or levels of strategy formulation, each with a different focus, which need to be dealt with in the formulation phase of strategic management. The three sets of recommendations must be internally consistent and fit together in a mutually supportive manner that forms an integrated hierarchy of strategy [7] (Figure 6).

- Corporate level strategy:
  1. deals with broad decisions about the organization's scope and direction,
  2. directs achieving stability to varying degrees of growth,
  3. directs portfolio of lines of business,
  4. directs allocation of resources and manage capabilities.
- Business or competitive level strategy:
   involves deciding how the company will compete within each line of business (LOB) or strategic business unit (SBU).
- Functional strategy:
   refers to localized and shorter horizon strategies which deal with how each functional area and unit will carry out its functional activities to be effective and maximize resource productivity.

*Strategy Implementation.* The translation of formulated strategy in line with the organization's mission and goals is the function of strategy implementation. The strategy implementation is only successful when there is stability between strategy and the organizational dimensions such as organizational structure, reward structure, and resource-allocation process.

**Figure 7:**
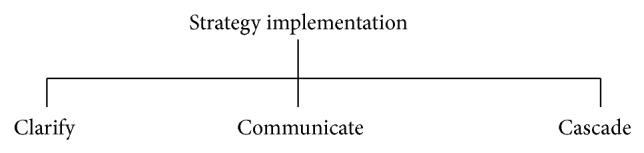
Strategy implementation.

To successfully execute an organization's strategy, it must be the focus of every person in that organization [1]. Scott Edinger derived three Cs to implement a strategy successfully (Figure 7).

**Figure 8:**
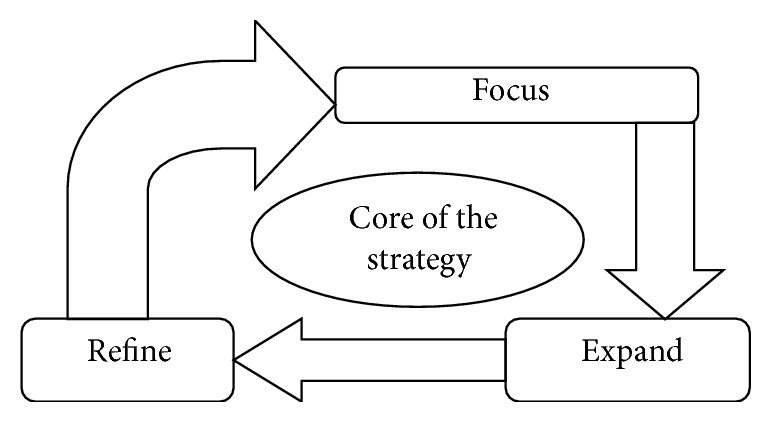
Strategy clarification [8].

*Strategy Clarification.* First step of the implementation is making the people understand what role they are playing in the process. If there is no clarification in the strategy, implementation will not meet out the organization's expectation. Scott Edinger states that “all too often, strategies are expressed as high-level statements that resonate with board and executive levels but fall flat with midlevel and frontline personnel (Figure 8).”

*Strategy Communication.* The key of implementation process is the powerful communication of the essence of the strategy. Scott Edinger says “discussions need to occur at each level, translating the organization's strategy to understandable and contextualized sound bites, which connect to the work of individuals. In short, communicating the strategy provides the “connective tissue” throughout the organization that helps people understand the big picture (Table 1).”

*Strategy Cascade.* The bulk of the work in implementing strategy is done at this stage. It is the team meetings, the one-on-one coaching, the process improvements, the customer meetings, and the responses to the market that, in alignment with an organization's strategy, can make a tremendous difference for an organization.

Jeroen De Flander states that “when cascading company's strategy, break down the objectives into smaller chunks for the next organizational level. The process stops at the smallest unit level, often teams. In the end, the size of organization will define the size of the cascade” [9]:

1. macroalignment,
2. microalignment.

*Macroalignment.* Macroalignment is a process stating that everything from the level below (strategy, initiatives, objectives, etc.) should add up exactly to the level above, without any overlaps [9]. The challenges of macroalignment are the complex matrix of the responsibilities and the strategy execution accountabilities.

*Microalignment.* Microalignment is the process that needs to balance goals and perspectives. The four most traditional perspectives are people, finance, internal process, and the customer.

Apart from balancing the macro- and microlevels, it is selecting key performance indicator (KPI) to track the objectives and defining appropriate targets.

*Strategy Evolution.* “Strategic evaluation is a way for businesses to evaluate the health and productivity of their company and their future endeavors. Typically, strategic evaluations attempt to see past the obvious factors that influence short-term plans and seek a more dynamic study of the trends that will dictate the future success or failure of the company. Like a chess match, strategic evaluation succeeds when companies are able to accurately analyze and predict several moves ahead into the future, in order to best tailor their present policies” [11]. Strategic evaluation emphasizes that evaluation of design decisions should be driven by the strategic value of the information they will provide for solving social problems [12].

The Anderson School of UCLA states four general principles of strategy evolution which are consistency, consonance, competitive advantage, and feasibility. If the strategy fails to meet any one of the above criteria, there should be a change in the strategy (Table 2).

*Consistency.* The strategy must not present inconsistent goals and policies. But in reality many strategies have been explicitly formulated but have evolved over a time lack of justification. Even strategies that are the result of formal procedures may contain compromise between two power groups. 

*Consonance.* An adoptive response to the external environment and to the critical changes occurring with the environment represents consonance of the strategy. 

*Competitive Advantage.* The strategy must provide for the creation and maintenance of a competitive advantage in the selected area of the activity.

*Feasibility.* The strategy must neither overtax available resources nor create unsolvable subproblems.

#### 5.1.3. Decision Making

Decision making is ongoing process of evaluating situations and problems, considering alternatives, making choices, and following them up with the necessary actions. The entire process is dependent upon the right information being available to the right people at the right times. According to Trewatha and Newport, “decision making involves the selection of a course of action among two or more possible alternatives in order to arrive at a solution to a given problem.”

Decision making is necessary in planning, organizing, directing, controlling, and staffing [13].

**Figure 9:**
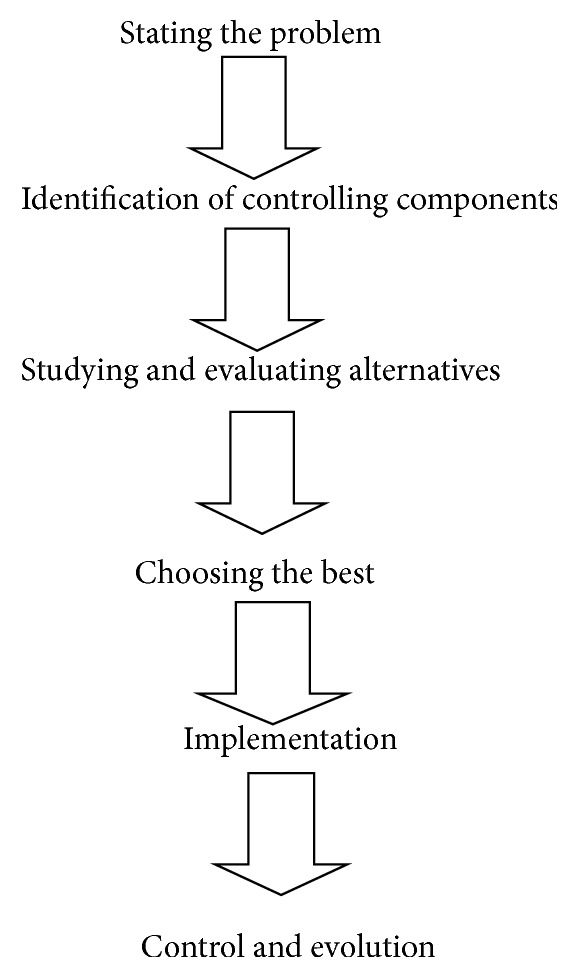
SISCIC decision making process.

SISCIC is a six-step decision making process which is described as shown in Figure 9 and Table 3.

## 6. Conclusion

Management process directs employees carrying out their duty in an ethical way. It focuses on proactive approach which enables the organization to grasp every opportunity that is available in the market. Hence this component framework implementation could be supporting business processes and ensuring business continuity; therefore, from an organizational perspective, as a replacement system, it has not failed totally.

## Figures and Tables

**Table 1:** Strategy communication methods and influencing factors.

Communication methods	Influencing factors
(1) Simple but meaningful	Strategic oriented information to help employees connect their day to day efforts towards the organization's mission and goal.

(2) Build behavior on market and customer insights	Encourage the team to develop department specific responses and generate new ideas.

(3) Inspire	The content should demonstrate progress against goals, showcase benefits to customers, and be presented in a way that gets attention and signals importance.

(4) Educate	To educate your teams most effectively on the validity of your strategy and their role in successful execution and make sure you provide job-specific tools with detailed data that they can customize and apply in their day-to-day responsibilities.

(5) Reinforce	Need to repeat the message in order to increase understanding, instill belief, and lead to true change overtime. These reinforcing messages need to come in a variety of tactics, channels, and experiences and we've highlighted some approaches below. Ultimately, they serve to immerse employees in important content and give them the knowledge to confidently connect to the strategy.

(6) Integrate	Integrate regular communications into employee's daily routines through detailed planning against the messages mapped in Inspire/Educate/Reinforce framework.

“Eight ways to communicate your strategy more effectively” by Everse HBR August 2011 [10].

**Table 2:** Strategy evolution principle and influencing factors.

Principle	Influencing factors
Consistency	(1) The problems in coordination and planning continue despite changes in personnel, due to inconsistencies of the strategy. (2) Organizational conflict and interdepartmental bickering also indicate problems of inconsistency. (3) Strategy in between organizational goals and the value of management group. Though inconsistency in this area is more of a strategy formulation than in the evolution of the strategy. Still it can arise; then the future direction of the business requires changes in managerial values.

Consonance	(1) The generic aspect of the strategy deals with the business adoptability with the environment. This represents the basic mission or the scope of the business. This analysis depends on the changing economic and social conditions over a time. (2) The competitive aspect of the strategy deals with the competition of the other firms who are also trying to adopt. This deals with the competitive edge. This analysis depends on the differences across the firm. (3) Sales growth is the success indicator of the generic strategy and the increased corporate worth is the success indicator for the competitive strategy.

Competitive advantage	(1) Superior skills: the skills that compose advantages are organizational rather than individual skills. Coordination and collaboration of the individual specialist are built through the interplay of investment, learning, and work. (2) Superior resources: the resources that include patents, trade mark rights, physical assets, and working relationship with suppliers and distribution channels. (3) Superior position: the best supply position involves supplying valuable products to the intensive buyers and the worst supply position involves supplying less valuable products to well-informed price sensitive buyers.

Feasibility	(1) This study assesses the problem solving abilities and or special competencies required by the strategy. (2) This study assesses the degree of coordinative and integrative skill necessary to carry out the strategy. (3) This study assesses the fact that “the strategy challenges and motivates key personnel and it is acceptable to those who must lend their support.”

**Table 3:** SISCIC process—steps and influencing factors.

Steps	Influencing factors/components
Stating the problem	(1) *Low profits* ^**^ due to *poor market research* ^*^ (2) *High costs* ^**^ due to *poor design process* ^*^ (3) *Low morale* ^**^ due to *lack of communication* ^*^ between the management and the team (4) *High employee turnover* ^**^ due to *rate of pay to low* ^*^ (5) *High rate of absentees* ^**^ due to the fact that *employees believe that they are not valued* ^*^

Identification of limiting components	(1) Information (2) Time (3) Equipment (4) Supplies (5) Personnel

Studying and evaluating alternatives	(1) Feasibility (2) Realistic (3) Effectiveness (4) Consequences (5) Cost-benefit^***^

Choosing the best	(1) Cost (2) Risk (3) Probability of success

Implementation	(1) Defining the role of employees and make them to understand. (2) Presenting the program and the procedures. (3) Framing the rules and the policies.

Control and evolution	(1) Feedback (2) Comparison of decision and action. (3) Measuring the deviation. (4) Removing deviation.

^*^Denotes the *problem*.

^**^Denotes the *cause*.

^***^ *Cost-benefit analysis* is a systematic process for calculating and comparing benefits and costs of a project or decision.
